# Supplementary material for: Influence of Drought and Heat Stress on Mineral Content, Antioxidant Activity and Bioactive Compound Accumulation in Four African Amaranthus Species
Source: Plants (Basel). 2023 Feb 20;12(4):953. doi: 10.3390/plants12040953 (PMC9966708; doi:10.3390/plants12040953)
Supplement: Supplementary file 1 [file plants-12-00953-s001.zip › plants-2113062-supplementary.pdf]

Appendix SA: Total phenolic and flavonoids compounds contents detected in Methanol (MeOH) and aqueous leaves extracts of *Amaranthus* species grown under WW and DI conditions (µg/g). ND (not detected). Data are shown as mean ± standard deviation and different letters in the same row indicate significant differences ($p \leq 0.05$).

Compound	*A. caudatus*				*A. hypochondriacus*				*A. cruentus*				*A. spinosus*			
	MeOH WW	MeOH DI	Aqueous WW	Aqueous DI	MeOH WW	MeOH DI	Aqueous WW	Aqueous DI	MeOH WW	MeOH DI	Aqueous WW	Aqueous DI	MeOH WW	MeOH DI	Aqueous WW	Aqueous DI
**Catechin**	7,2 ± 0,006a	7,2 ± 0,028a	7,2 ± 0,003a	7,2 ± 0,004a	7,2 ± 0,012b	7,2 ± 0,004a	7,2 ± 0,002a	7,21 ± 0,009b	7,2 ± 0,008a	7,21 ± 0,011b	7,2 ± 0,001a	7,2± 0,004a	7,2 ± 0,073b	7,2 ± 0,003a	7,2 ± 0,002a	7,2 ± 0,002a
**P-hydrobenzoic acid**	33,38 ± 7,01a	ND	ND	ND	ND	ND	ND	ND	ND	ND	ND	ND	ND	ND	ND	ND
**Caffeic acid**	31,82 ± 2,87c	20,12 ± 0,43a	27,523 ± 4,21b	30,45 ± 0,65c	ND	ND	254,39 ± 4,32d	1064,41 ± 8,21e	ND	ND	ND	ND	ND	ND	ND	ND
**Vanillic acid**	36,89 ± 3,06c	17,77 ± 5,23b	48,06 ± 15,63d	37,01 ± 0,40c	ND	ND	8,07 ± 1,24a	6,09 ± 1,27a	ND	ND	ND	ND	ND	ND	ND	ND
**Rutin**	179,1 ± 10,45g	353,1 ± 15,68k	10,18 ± 0,56c	8,23 ± 0,027b	159,9 ± 14,29f	267,4 ± 9,26j	8,07 ± 0,27ab	14,48 ± 0,47d	143,1 ± 0,34e	206 ± 10,61h	11,5 ± 0,72c	6,82 ± 0,12a	139,8 ± 16,24e	215,5 ± 61,00i	27,01 ± 0,30e	15,08 ± 0,89d
**P-coumaric acid**	31,98 ± 6,31c	8,12 ± 0,54a	157 ± 22,22e	118,87 ± 6,38d	ND	ND	33,29 ± 1,99c	24,46 ± 2,96b	ND	ND	ND	ND	ND	ND	ND	ND
**Isoquercetin**	62,1 ± 7,14a	161 ± 10,006c	ND	ND	106,2 ± 8,58b	ND	ND	ND	ND	ND	ND	ND	ND	ND	ND	ND
**Quercetin**	151,5 ± 7,51f	286,4 ± 18,80i	ND	ND	224,5 ± 25,32h	187,8 ± 25,87g	ND	ND	69,4 ± 10,83d	121,4 ± 21,41e	13,96 ± 1,431a	ND	ND	36,21 ± 1,63b	51,9 ± 0,92c	ND
**Kampferol-3-O-rutinoside**	ND	22,23 ± 2,66c	ND	ND	22,71 ± 5,10c	24,59 ± 2,21d	ND	ND	14,52 ± 2,21b	22,72 ± 3,94cd	ND	ND	36,12 ± 9,13e	12,54 ± 0,87a	16,416 ± 0,34b	ND
**Sinapic acid**	15,2 ± 3,93b	11,31 ± 1,97a	ND	ND	ND	ND	ND	ND	ND	ND	ND	ND	ND	ND	ND	ND
**Ferulic acid**	21,3 ± 4,97c	25,03 ± 5,26d	173,5 ± 13,81i	121,31 ± 6,26h	ND	ND	72,1 ± 19,50f	82,1 ± 5,94g	ND	16,3 ± 1,19a	42,65 ± 8,20e	ND	ND	ND	19,55 ± 1,88b	15,591 ± 2,24a
**Gallic acid**	ND	ND	ND	ND	ND	ND	ND	ND	ND	ND	ND	ND	ND	ND	ND	ND
**Coumarin**	ND	ND	ND	ND	ND	ND	ND	ND	ND	ND	ND	ND	ND	ND	ND	ND
**Ellagic acid**	ND	ND	ND	ND	ND	ND	ND	ND	ND	ND	ND	ND	ND	ND	ND	ND
